# A MOOC as an immediate strategy to train health personnel in the cholera outbreak in Mexico

**DOI:** 10.1186/s12909-018-1215-1

**Published:** 2018-05-16

**Authors:** Laura Magaña-Valladares, Cynthia Rosas-Magallanes, Alejandra Montoya-Rodríguez, Guillermo Calvillo-Jacobo, Celia Mercedes Alpuche-Arande, Sebastían García-Saisó

**Affiliations:** 1 Association of Schools and Programs of Public Health (ASPPH), 1900 M Street NW, Suite 710, Washington DC, 20036 USA; 2 Instituto Nacional de Salud Pública, Universidad 655, Col. Santa María Ahuacatitlán, C.P. 62100 Cuernavaca, Morelos Mexico; 3 Centro de Investigación Sobre Enfermedades Infecciosas, Universidad 655, Col. Santa María Ahuacatitlán, C.P. 62100 Cuernavaca, Morelos Mexico; 4 Secretaría de Salud, Homero 213, piso 12, Col. Chapultepec Morales, 11570 Ciudad de México, Mexico

**Keywords:** MOOC, Cholera, Epidemiological emergency, Health training

## Abstract

**Background:**

In September 2013, two cases of cholera were reported in Mexico; 1 week later, a new outbreak was reported in the Huasteca region of Hidalgo. Upon the determination that the diagnostic and therapeutic interventions implemented by health personnel overlooked predefined procedures, the National Institute of Public Health, in coordination with the Ministry of Health, immediately designed the massive open online course “Proper cholera containment and management measures” to strengthen and standardize basic prevention and control practices.

**Methods:**

During the first 5 months, 35,968 participants from across the country finished the course: medical and nursing personnel, health promoters, and hospital staff. To understand the magnitude of the data, an analysis was performed to calculate the MOOC coverage, and multiple linear regression models were generated to relate the score earned in the course to the characteristics of the participants. In addition, a qualitative analysis was performed to identify the dissemination of information, technological barriers, and feedback on course design. A total of 17% of participants were from the state where the outbreak originated, and 33.5% were from its neighboring states.

**Results:**

This study shows that the need for information is greater when an emergency occurs, and the involvement of the authorities increased the extent of the training response.

**Conclusion:**

A MOOC can be a useful training strategy to prepare personnel for emergency situations.

## Background

In September 2013, the National Epidemiological Surveillance System reported two cases of cholera in Mexico City classified in the same serogroup, which were different from those circulating in the country, with strains similar to those found in Haiti, Dominican Republic, and Cuba. One week later, four new cases were identified, and later that year, a total of 175 cases were confirmed in the Huasteca region of Hidalgo, inhabited mainly by Otomi indigenous people [1], located approximately 121 km from Mexico City.

The need for rapidly training health personnel emerged after the determination that the diagnostic and therapeutic interventions implemented by the health personnel differed significantly in similar patient cases. A team of epidemiological surveillance experts from the Ministry of Health made a diagnosis about the care services of health professionals in the region and detected that some health personnel ignored prescribed procedures in both diagnosis and treatment and, in some cases, introduced errors in both aspects. Therefore, immediately implementing a massive process of strengthening, updating, and standardization of knowledge and basic practices of disease prevention, control, and treatment was considered necessary. Faced with this challenge, the Ministry of Health assembled a committee of experts from renowned health, social security, nutrition, epidemiology, prevention, disease control, and technology development institutions to design and conduct the massive open online course (MOOC) titled “Proper cholera containment and management measures”.

It was decided to create a MOOC because it was the most efficient way to reach as many people as possible in a small period of time despite the low rates of terminal efficiency that the evidence presents in regard to these courses [2].

The design of the MOOC was founded on the competency-based educational model of the National Institute of Public Health (INSP in Spanish) and was launched for all healthcare professionals of Mexico from different sectors. The Ministry of Health (SSA in Spanish) sent official letters to its state heads of education for to urge the entire healthcare workforce to complete the course as soon as possible.

MOOCs are a valuable tool to distribute educational content through the Internet [3], with unlimited and open registration, and grounded in the theory of connectivism. This approach, developed by George Siemens, differs from behaviorism, cognitivism, and constructivism by fostering a close link between learning and Information and Communications Technology (ICT) [4]. The significant volume of participants that are gathered, the broad geographic reach, and the low unit cost are some of the positive attributes of MOOCs. Furthermore, MOOCs are designed under self-management systems, which enhance student interaction with content and automate learning activities.

The present article addresses the experience of designing and conducting the MOOC “Proper cholera containment and management measures” with the objective of analyzing the coverage, function, and feedback on the design of the course and key factors for its effectiveness to confront an emergency situation. This strengthens the studies that have been carried out on education in a state of emergency, which refers to quality learning opportunities in crisis situations, including public health emergencies [5–7].

## Methods

The MOOC, an educational format chosen for its ability to reach across the country and being self-directed, had the following four main topics: *a)* the concept of cholera and prevention procedures, *b)* diagnosis, *c)* treatment, and *d)* key aspects to conduct a timely epidemiological surveillance.

The MOOC was developed by a transdisciplinary group of academics and health officials who determined the competencies that health personnel require to adequately confront the cholera outbreak. A team of instructional designers created the learning activities based on the principles of andragogy [8], particularly meaningful and practical knowledge acquisition, and following the learning cycle through experiences that lead to reflection, conceptualization, and implementation of the acquired knowledge in real life situations. The development of the course took the INSP’s production and design team 8 days of full time work. The course was available in any device with internet access as well as with an offline version.

A qualitative and quantitative approach was used for its analysis.

The quantitative component focused on the analysis of the geographical, temporal and institutional coverage of the course and the relationship between sociodemographic characteristics (age, gender, marital status, affiliation, and profession) and the score earned in the course.

For the qualitative component, semi-structured interviews were conducted with managers and operational staff with their informed consent to identify the actions they implemented to promote the course, understand the management and availability of technology among the course attendees, and obtain feedback on the design and content functionality. Based on the information from the state with the largest share of participants after the state where the outbreak started, data were collected from 8 focus groups consisting of state health managers (including the Ministry of Health), Jurisdiction officials and hospital personnel.

The MOOC coverage in healthcare units was calculated by dividing the number of attendees registered in the course by the number of staff working in the primary care units in each state, according to the National Health Information System records. The geographical coverage was estimated for 6 geographic regions (Table 1), defined according to their proximity to Hidalgo state, where the first cholera outbreak occurred. Temporal coverage was defined as the number of attendees per week throughout the period when the course was available.

**Table 1:** Sociodemographic characteristics of participants

Characteristics		
Age, mean (SD)	35,934	36.40(10)
Score, mean (SD)	35,934	85.6(9.4)
	N	%
Women	35,934	65.3
Profession *N* = 34,506		
Physician	15,992	46.4
Nurse	12,929	37.5
Healthcare promoter	612	1.8
Other healthcare Professional	2517	7.3
Others	2456	7.1
Affiliation *N* = 35,753		
IMSS	14,151	39.6
ISSSTE	449	1.3
SSA	19,320	54.0
PEMEX, SEMAR, SEDENA	1093	3.1
OTHERS	740	2.1
Participation by geographic region N = 35,934		
Geographic region 1	6135	17.07
Geographic region 2	12,041	33.51
Geographic region 3	7077	19.69
Geographic region 4	4460	12.41
Geographic region 5	2725	7.58
Geographic region 6	3496	9.73

Geographic region 1. Hidalgo

Geographic region 2. Veracruz, Mexico State, Tlaxcala, San Luis Potosi, Queretaro and Puebla

Geographic region 3. Oaxaca, Morelos, Guanajuato, Michoacan, Guerrero, Federal District and Tamaulipas

Geographic region 4. Sonora, Durango, Coahuila, Sinaloa, Baja California, Chihuahua and Nuevo Leon

Geographic region 5. Zacatecas, Colima, Aguascalientes, Jalisco and Nayarit

Geographic region 6. Quintana Roo, Chiapas, Campeche, Yucatan and Tabasco

The main sociodemographic characteristics of participants were described using the mean, standard deviation, and percentages. Multiple linear regression models were fitted with fixed effects by geographic region, with the score as the dependent variable and gender, age, profession, and affiliation as independent variables to evaluate the relationship between the score received and the characteristics of the participants.

Transcripts from the focus groups were coded in Atlas.ti 7.0 for qualitative analysis. Coding was based on four families of codes related to the actions to implement the virtual course, training strategies prior to the course, management and availability of technology among the participants, and feedback on the design and functionality of the content.

## Results

### Terminal efficiency

During the first 5 months after the beginning of the course, 40,002 participants were registered; 4379 (9.9%) “no shows” and 35,968 participants finished the course. The terminal efficiency was 82.5% as shown in Table 2,

**Table 2:** Terminal efficiency

Health professionals staff	Enrolled participants	“No shows participants”	Participants who completed the MOOC^a^	Participants who accredited the MOOC (score higher than 7)	Participants who did not accredit the MOOC
966,083	40,002	4379 (9.9%)	35,968 (89.9%)	33,006 (82.5%)	2962 (7.4%)

^a^We chose these participants as the population in our analysis

### Sociodemographic characteristics of the participants

During the first 5 months after the beginning of the course, 35,968 participants from the 31 states and the capital of Mexico finished the course. More than half were women (65.3%) aged from 15 to 89 years, with a mean age of 36 years. Most attendees were physicians (46.4%) and nurses (37.5%), and the remaining 9.1% were either healthcare promoters or provided other professional services. Regarding affiliation, 54% worked for the Ministry of Health, 39.6% worked for the Mexican Social Security Institute (IMSS in Spanish), and 6.5% worked for the Institute for Social Security and Services for State Workers (ISSSTE in Spanish), Mexican Petroleums, Navy Department, Department of National Defense and other government institutions. The distribution of participants per geographic region showed that 17% were from Hidalgo state, where the outbreak started, 33.5% were located in neighboring states to the outbreak site (geographic region 2), and 19.7% were located in states of the 3 geographic regions farthest from the outbreak (Table 1).

### Coverage

State coverage (Table 3) ranged from 0.3 to 36% of all healthcare personnel in the states. Hidalgo, the state where the outbreak started, recorded the highest coverage rate, followed by Quintana Roo, Zacatecas, and Veracruz. When considering the institutional affiliation of the attendees, the Hidalgo State Department of Health reached 53.3% participation, whereas Quintana Roo reached up to 25% participation. In absolute terms, the IMSS ranked second in the number of participants but outperformed the SSA in 20 states when calculating the percentage of coverage. In turn, Zacatecas state achieved the highest percentage of coverage in the IMSS, with just over 30%.

**Table 3:** Coverage by sector and state

Federal entity	IMSS Coverage	ISSSTE Coverage	SSA Coverage	Total Coverage
Hidalgo	10.7%	1.8%	53.3%	36.0%
Quintana Roo	5.6%	7.1%	25.0%	15.2%
Zacatecas	30.3%	0.4%	3.8%	11.9%
Veracruz	6.1%	1.2%	12.0%	9.3%
Estado de México	4.3%	2.2%	11.6%	7.9%
Oaxaca	19.3%	0.6%	4.2%	7.5%
Durango	11.6%	0.7%	0.6%	5.0%
Morelos	3.3%	1.1%	6.7%	4.7%
Sonora	6.1%	0.6%	1.1%	3.7%
Chiapas	11.0%	0.2%	0.4%	3.3%
Guanajuato	4.7%	0.2%	1.9%	2.9%
Michoacán	1.7%	0.2%	3.7%	2.8%
Guerrero	2.2%	1.1%	2.8%	2.7%
Coahuila	5.1%	0.0%	0.1%	2.7%
Tabasco	6.7%	0.0%	0.2%	2.6%
Campeche	4.6%	0.8%	0.1%	2.6%
Aguascalientes	2.4%	0.2%	2.3%	2.2%
Sinaloa	4.5%	0.2%	0.2%	2.1%
Yucatán	2.2%	0.5%	1.9%	1.9%
Tlaxcala	4.3%	0.3%	0.8%	1.9%
Chihuahua	1.1%	0.4%	2.2%	1.6%
Colima	0.8%	0.2%	0.7%	1.6%
Baja California	1.9%	1.4%	1.1%	1.5%
Jalisco	0.8%	0.4%	2.2%	1.4%
Distrito Federal	2.5%	0.1%	0.4%	1.3%
Tamaulipas	0.4%	0.3%	0.1%	0.7%
Nayarit	0.3%	0.5%	0.8%	0.6%
Nuevo León	0.8%	0.1%	0.3%	0.6%
San Luis Potosí	0.7%	0.2%	0.2%	0.5%
Querétaro	1.9%	0.3%	0.1%	0.5%
Puebla	0.3%	0.0%	0.1%	0.3%

The results showed that the coverage was higher when senior officials in the healthcare system were involved in launching the online course.

“…The leadership of our senior officials facilitated conducting the course on cholera and encouraged all stakeholders to attend the course, from managers to the custodial staff. This also strengthened the use of technology by the staff...”.

**Fig. 1:**
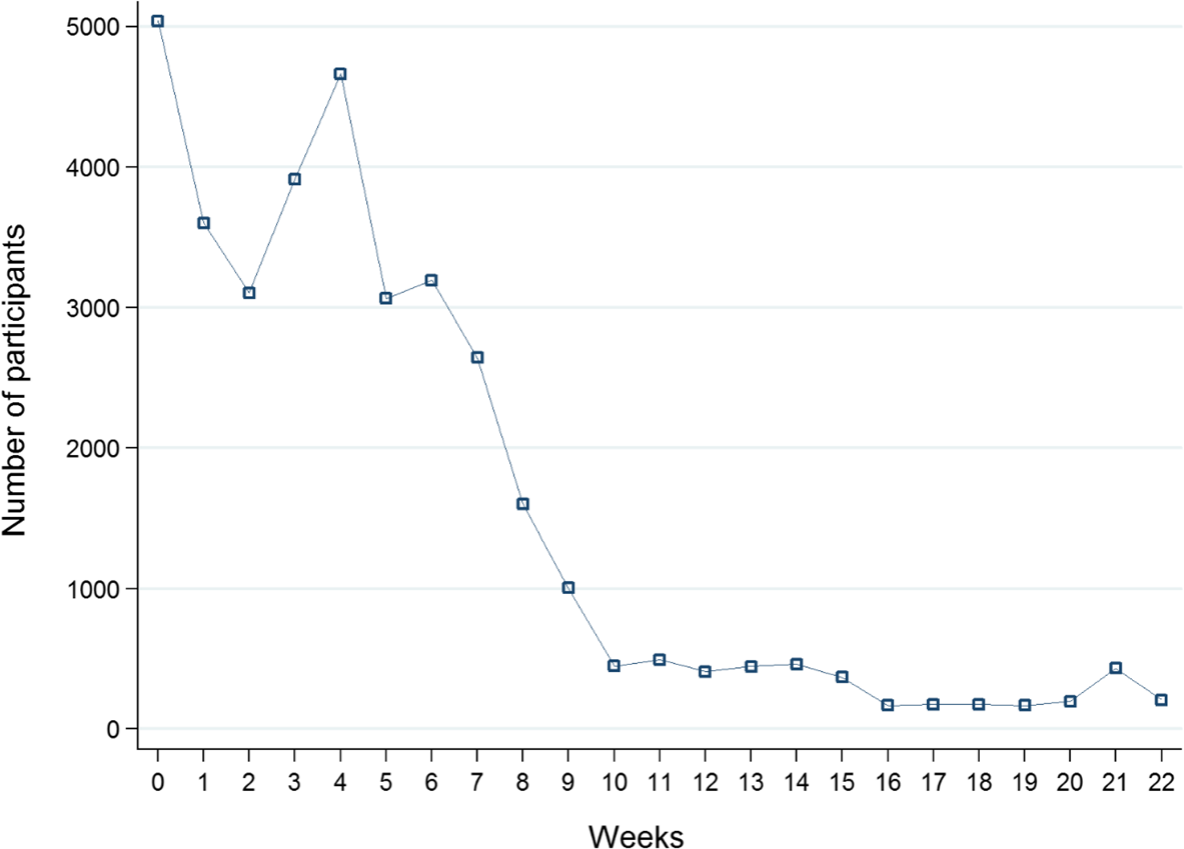
Course participation trend

During the first week, 5000 people enrolled, and over the first 7 weeks, the weekly number of enrollments ranged from 3000 to 5000 people (Fig. 1: Course participation trend). This occurred until mid-December, when the course registration trend decreased significantly, remaining at a constant rate of 250 to 500 enrollees per week.

**Fig. 2:**
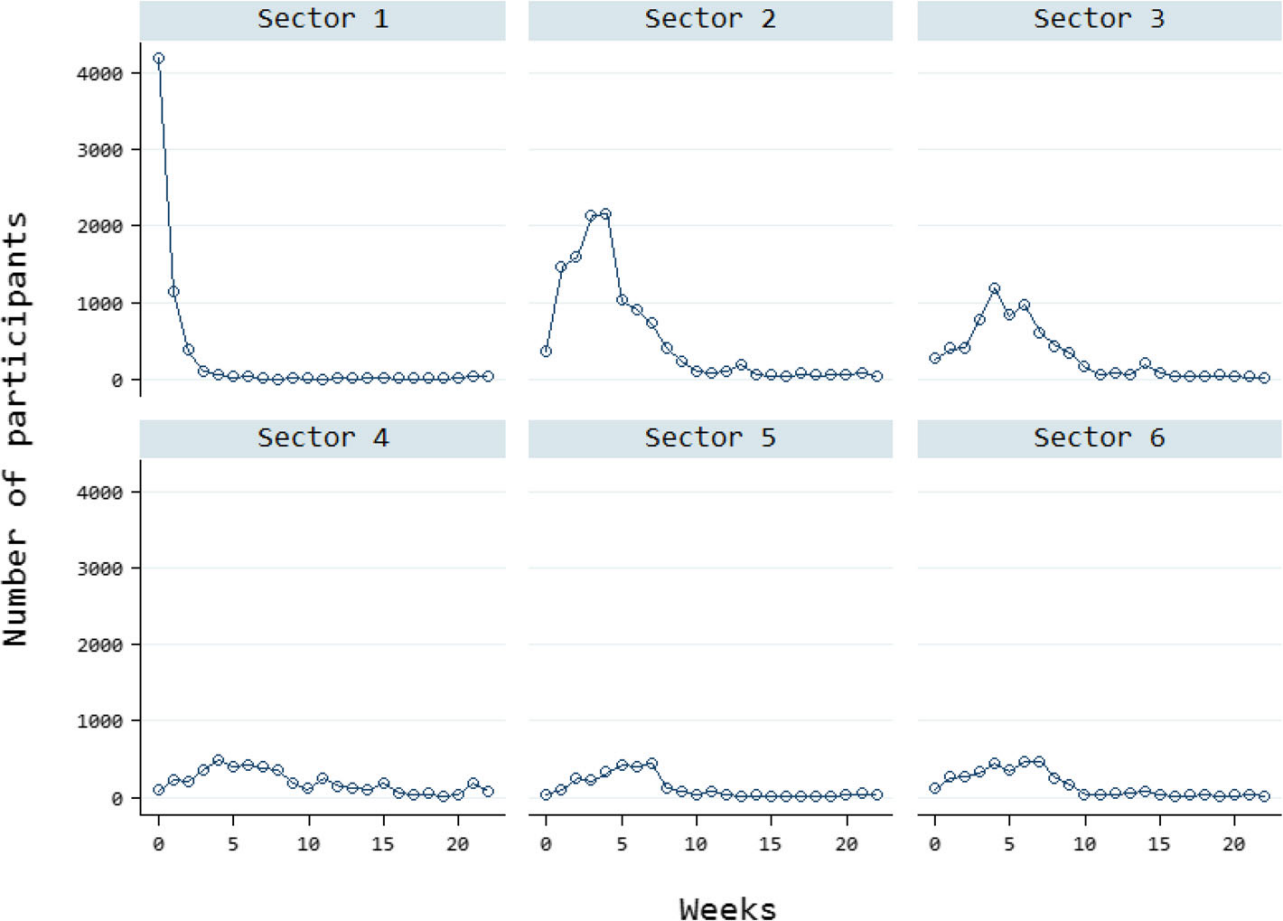
Course participation by sector

Figure 2 (Trend of participation in the course on proper cholera containment and management measures per geographic region) shows that the participation trend of healthcare providers differed according to the geographic region and was much more active during time periods and places closest to the outbreak. In geographic regions 1, 2, and 3, maximum participation occurred during the first 3 weeks of training, when the outbreak started, whereas steady enrollment was observed throughout the training period in the remaining geographic regions.

### Results from the learning evaluation

The overall average score among participants was 85.6 from a maximum score of 100; however, 6.29% of participant’s scores was not high enough for accreditation (scores below 70). Multivariate regression models showed slightly differences in the average scores between men and women, with respect to participants’ age and profession (less than one point of difference in average scores). However, there were more differences in the scores among health institutions. After controlling sex, age, profession and geographic region, ISSSTE healthcare providers and other health affiliations achieved lower average scores than those achieved by the IMSS health staff (5.6 and 2.16 respectively; *p* < 0.05, Table 4). The terminal efficiency, 82.5% (33,006 participants) as the course had an open modality only those who wanted their certificate were registered and answered the final evaluation. This evaluation consisted of four clinical cases that had to be answered by 20 multiple choice questions and 10 true and false questions. For the evaluation criteria in the answers, a maximum score of 100 was considered while the minimum score consisted of 70 points.

**Table 4:** Multiple linear regression model for the association between scores achieved^a^ on the MOOC and the participant’s characteristics

	Β	CI_95%_	
Sociodemografics			
Men^b^	–	–	–
Women	0.740*	(0.41,	0.107)
Age (years)	0.051*	(0.04,	0.06)
Profession			
Physician^b^	–	–	–
Nurse	−0.07	(−1.17	1.03)
Healthcare promoter	−0.45	(0.49	1.40)
Other healthcare professionals	−01.04*	(−1.98	−0.09)
Affiliation			
IMSS^b^	–	–	–
ISSSTE	−5.62*	(−8.19,	−3.06)
SSA	0.4	(−0.57,	1.38)
PEMEX, SEMAR, SEGOB, SEDENA	−0.29	(−2.54,	1.95)
OTHERS	−2.16*	(−4.03,	−0.29)
Geographic regions			
Geographic region 1^b^	–	–	–
Geographic region 2	−1.41*	(− 1.73,	− 1.08)
Geographic region 3	− 1.34*	(− 2.63,	−0.05)
Geographic region 4	−0.13	(−1.34,	1.09)
Geographic region 5	0.3	(−0.88,	1.48)
Geographic region 6	−1.94*	(−2.54,	− 1.34)

Geographic region 1. Hidalgo

Geographic region 2. Veracruz, Mexico State, Tlaxcala, San Luis Potosi, Queretaro and Puebla

Geographic region 3. Oaxaca, Morelos, Guanajuato, Michoacan, Guerrero, Federal District and Tamaulipas

Geographic region 4. Sonora, Durango, Coahuila, Sinaloa, Baja California, Chihuahua and Nuevo Leon

Geographic region 5. Zacatecas, Colima, Aguascalientes, Jalisco and Nayarit

Geographic region 6. Quintana Roo, Chiapas, Campeche, Yucatan and Tabasco

^a^Score expressed on a 0–100 scale

^b^Reference categories

* *P*-value<.05

### Feedback on the course

The participants characterized the graphic design of the course as a resource that facilitated and encouraged their participation.

“…at first, we thought that the course only involved readings. However, we soon realized that it contained other educational materials with innovative designs...”.

Trainee nurse 2. Chetumal General Hospital, 2014.

Participants also shared encouraging comments about the instructional design, which they characterized as functional, easy to handle, dynamic, and full of well-structured content, presented with simplicity, facilitating learning and feedback.

“…The course was highly educational for all the hospital staff, even for janitors of hospitals and health centers. It taught them to take preventive health measures required to prevent and even reverse the epidemic...”. Epidemiologist from Jurisdiction # 1, Chetumal, Quintana Roo, 2014.

During the qualitative research phase, respondents mentioned old age as an impediment to the use of technology. Fortunately, this obstacle was overcome thanks to peer support and workplace facilities, particularly in healthcare centers, hospitals, and teaching areas of health jurisdictions, where a type of companionship and mutual support was generated, which ultimately favored the submission of evaluations. Examples include the group of janitors who took the course at the home of the Head of Administration in Chetumal, and the healthcare professionals who took the MOOC at the Office of the Head of Education in Cancun.

It was also reported that printouts from the MOOC were used to conduct the course in a workshop format in communities without electricity. Furthermore, different graphic and content elements were used to provide information to the population about the disease. This work was primarily performed by interns, who, in turn, recommended including downloadable support files.

## Discussion

The terminal efficiency of the course is far above from what the evidence suggests. However, since it is an open course, only those participants who actually wanted to obtain their certificate were registered. This is why it was possible for the terminal efficiency to be higher than stipulated. The results of our analysis put into perspective the great reach and impact MOOCs may achieve in a public health emergency. This strategy was so effective in Mexico that it has been replicated to combat other disease outbreaks that have occurred in the country, including dengue, malaria, chikungunya, and Zika, among others.

The considerable participation in the MOOC is related to the emergency; greater participation and interest was observed at the beginning of the outbreak, which remained steady for 7 weeks and then decreased once the situation in the country was controlled. This indicates that humans, when facing an emergency, have increased interest in learning, but their interest in learning decreases when the threat is controlled [6, 7]. A MOOC based on andragogy, the learning cycle, and a competency-based constructivist focus results in key academic achievement and good perception and high levels of satisfaction among the participants.

The states of geographic region 2, which borders the state in which the outbreak occurred, were expected to record greater coverage, but this was not the case. Therefore, it is surprising that Quintana Roo had the highest participation rate despite its geographical location away from the epidemiological focus (region 6), demonstrating that in addition to the perceived need for training, political will, monitoring, and support of the authorities are essential to increase participation. In this state, the authorities at all levels were involved and attended the MOOC in teams, encouraging all staff to take the course.

The student enrollment data throughout the 5 months of the study allowed us to conclude that the MOOC was very helpful to spread knowledge to broad healthcare systems in very short periods of time. The speeds at which the students took the course, considering the classification of states by geographic regions, shows a consecutive trend consistent with the proximity to or distance from the outbreak, regardless of coverage. The fastest reactions were observed in decreasing order in geographic regions 1, 2, and 3. This finding indicates that the need for information is greater when an emergency occurs [9, 10].

## Conclusions

The MOOC titled “Proper cholera containment and management measures” is a valuable example of an efficient strategy designed to standardize healthcare information and processes in cases of epidemiological emergency. The following were key elements of its effectiveness:

1. An educational design technologically supported by the competency-based and constructivist educational model. Each MOOC activity followed the learning cycle to motivate the students. This led to a mean score in the course of 85.6 among all participants;
2. Clear, simple messages with graphic elements and recreational activities demonstrating their effectiveness for different audiences and attending staff;
3. Peer support and local organization to ensure personnel involvement; and
4. The commitment of senior management. Once again, the results show that greater participation was achieved when senior management was involved.

For the research, it is needed to assess the impact of this training on the health indicators of each state, how they contained the outbreak, and the different average reaction time between participants who completed the MOOC and those who did not. For Mexico, this experience marks the beginning of a series of MOOCs that have been developed to immediately train health personnel to confront various emergencies.

One of the limitations of the study is that only the participants who completed the course (approved or failed) were taken into account in the quantitative analyzes, and “no shows” were not considered. A second limitation implies that there was not a specific strategy to minimize the cheating or sharing of answers among the participants. Therefore, this topic could imply a weakness of the course.

## Abbreviations

IMSS: Instituto Mexicano del Seguro Social (Mexican Social Security Institute)
INSP: Instituto Nacional de Salud Pública (National Institute of Public Health)
ISSSTE: Instituto de Seguridad y Servicios Sociales de los Trabajadores del Estado (Institute for Social Security and Services for the Sate Workers)
MOOC: Massive Open Online Course
SSA: Secretaría de Salud (Ministry of Health)
